# Joint Growth Trajectories of Trait Emotional Intelligence Subdomains Among L2 Language Learners: Estimating a Second-Order Factor-of-Curves Model With Emotion Perception

**DOI:** 10.3389/fpsyg.2021.720945

**Published:** 2021-09-13

**Authors:** Tahereh Taherian, Ali Mohammad Fazilatfar, Golnar Mazdayasna

**Affiliations:** Department of English Language and Literature, Yazd University, Yazd, Iran

**Keywords:** trait emotional intelligence, emotion perception, longitudinal study, parallel-process modeling, factor of curves modeling

## Abstract

The present study assessed the developmental dynamics of trait emotional intelligence (TEI) and its subdomains during English as a foreign language (EFL) learning in a longitudinal study. A sample of 309 EFL learners (217 females, 92 males) was used to assess the trajectories of the global factor of TEI and the parallel development of the TEI subdomains over 1 year in the context of the EFL classroom using parallel process modeling (PPM) and factor of curve modeling (FCM). Additionally, emotion perception (EP) was used as a distal outcome to investigate how growth parameters, including intercept and slope factors in a TEI-FCM, influence the distal outcome of EP. The results revealed that there was sufficient inter-individual variation and intra-individual trends within each subdomain and a significant increase over time across the four subdomains. Additionally, concerning the covariances within and among the subdomains of TEI, the PPM results revealed moderate to high associations between the intercept and slope growth factors within and between these subdomains. Finally, regarding the direct association of the global growth factors (intercept and slope) of TEI on EP, the results indicated that the intercept and slope of global TEI were associated with EP (γ_0_ = 1.127, *p* < 0.001; γ_1_ = 0.321, *p* < 0.001). Specifically, the intercepts and slopes of emotionality and sociability turned out to be significantly linked to EP (γ_03_ = 1.311, *p* < 0.001; γ_13_ = 0.684, *p* < 0.001; γ_04_ = 0.497, *p* < 0.001; γ_14_ = 0.127, *p* < 0.001). These results suggest the dynamicity of TEI during learning a foreign language are discussed in this study in light of the potential variables associated with TEI and its related literature.

## Introduction

One of the important abilities used to understand the emotions of others and oneself is emotional intelligence (EI), which can be operationalized as the level of a personality trait or as a developing ability. Thus, EI can be defined as an extensive range of individual differences that expresses the properties of the intelligence of an individual and their subjectively emotional experiences (Hughes and Evans, 2018). Furthermore, EI enables individuals “to manage their emotion for thinking and thinking for emotion management” (Dewaele et al., 2008, p. 912). It can be conceptualized based on different theoretical approaches (Petrides, 2010; Hughes and Evans, 2018). Among these, the trait approach (Petrides et al., 2016) defines EI as the self-rating of an individual regarding his or her emotional ability. Trait emotional intelligence (TEI) has been categorized into four main subdomains: well-being, emotionality, self-control, and sociability (Petrides and Furnham, 2000, 2001). Petrides et al. (2016) reported a comprehensive review of the application of TEI in versatile contexts. There is evidence highlighting the crucial role of TEI not only among the L1 users (Pekaar et al., 2020), but also among learners of a second or foreign language by enabling them to set up their communicative goals (Dewaele et al., 2008) and control their communicative anxiety (Dewaele et al., 2008; Li and Xu, 2019). Research has indicated that learners with high TEI experience more foreign language enjoyment (FLE) (Li and Xu, 2019; Li, 2020; Resnik and Dewaele, 2020; Li et al., 2021), are more confident in managing their own feeling (Oxford, 2017), and comprehend the emotions of their interlocutors (Oxford, 2017; Alqarni and Dewaele, 2020) better than those with low TEI.

Even though research has indicated that TEI is a pivotal antecedent to learning a new language, the basic processes that underpin its effects are yet to be explored (Pekaar et al., 2020). Research on EI has typically focused on the exploration of the construct at the global level over one or two time points. Such approaches, however, discard the interplay among the dimensions TEI and its temporal aspects (Pekaar et al., 2020). Consequently, these traditional research designs could not examine how TEI develops over time, how different dimensions of TEI interact and influence global TEI, and what the distal consequences of individual TEI growth are over time. In response, Pekaar et al. (2020) inspired researchers to take advantage of multilevel designs to capture the individual differences in TEI and its fluctuations across time.

Hence, to have a more comprehensive conceptualization of TEI, its developmental nature and the development of its subdomains should be taken into account; that is, more complicated models should be developed to identify the interactive effect of these subdomains and their effects on the global factor of TEI over time. With this in mind, we aimed to investigate both the primary growth factors of TEI and their covariations in order to explore the co-development of the different subdomains of TEI and the contribution of each subdomain to the global factor of TEI.

In addition, the model in this study was extended by including emotion perception (EP) as a distal outcome since studies in the realm of psychology of language learning have indicated that TEI is a pivotal construct for successful EP. In particular, EP is the ability to perceive and interpret the emotions of others precisely (Dewaele et al., 2019; Lorette and Dewaele, 2019; Alqarni and Dewaele, 2020). Years ago, Mayer and Salovey (1997) asserted that individuals with high TEI have a greater ability to recognize the emotions of other individuals *via* both facial expressions and their “own muscular and bodily sensations and social surroundings” (p. 10). This finding implies that individuals with high TEI can recognize consensual emotion content across diverse domains since they can recognize the thought processes of other individuals, and thus be able to come up with some universal rules for the detection of their emotions. Therefore, to test our hypothesis involving the role of TEI on the ability to perceive the emotions of others in the current study, EP was also incorporated into the factor of curve modeling (FCM) as a distal outcome, creating a conditional FCM.

## Review of the Related Literature

### Emotional Intelligence: Theories, Constructs, and Measurements

A number of EI models have been introduced in the field of psychology, but two of the more dominant models are the ability EI model (Salovey and Mayer, 1990) and the TEI model (Petrides, 2017). Although these two models have some similarities, they have some distinguishing differences. The first task measure for *ability EI*, which seems comprehensive and theory-based, was the Multifactor Emotional Intelligence Scale (MEIS) (Mayer et al., 1990). It was later revised by Mayer et al. as the Emotional Intelligence Scale (MSCEIT) (Mayer et al., 2002). Using a deductive approach, Salovey and Mayer (1990) introduced the model of ability EI with four aspects: (1) the ability to detect emotions precisely, (2) the ability to apply emotions to further thought, (3) the ability to comprehend emotions, and (4) the ability to regulate emotions. The primary implication of this model is that EI, similar to any other cognitive ability, could be fostered as a consequence of direct practice.

On the other hand, Petrides and Furnham (2000) proposed the TEI model, which captures the self-perceived or subjective emotional abilities of individuals and is estimated with a self-rated questionnaire. Cooper and Petrides (2010) defined TEI as “a constellation of emotional self-perceptions located at the lower levels of personality hierarchies” (Petrides et al., 2007, p. 449). Therefore, TEI is completely outside the cognitive ability domain and can be incorporated into the structural models of personality. Petrides (2017) argued that TEI deals with comprehension, not abilities or talent. A learner with a high TEI level is not necessarily flexible or adaptive and an individual with a low level of the construct is not necessarily inflexible or maladaptive since adaptive orientation relates to situational demands and contextual factors.

Trait emotional intelligence entails 15 facets categorized in to four main subdomains or dimensions: well-being, emotionality, sociability, and self-control (Petrides and Furnham, 2003). The dimension of well-being is represented *via* having self-confidence, pleasure, and feeling fulfilled with life (happiness), and to have an eye on the bright side of life (optimism). The self-control dimension refers to the abilities to regulate emotions (emotional regulation), not give in to demands (impulsiveness), and endure burden and manage stress (stress management). The emotionality dimension is related to the skill of considering the point of view of others (empathy), being certain about the emotions of an individual (emotional perception), interacting with other people with emotions (emotional expression), and sustaining satisfied personal relationships (relationships). Finally, the sociability dimension reflects the ability of an individual to affect the emotions of other individuals (emotional management), defend their own rights (assertiveness), and to create networks *via* social skills (social awareness).

Personality research has found evidence of the dynamicity of TEI over the years (Parker et al., 2005; Dave et al., 2021). The dynamicity of the construct is important to assess because it can provide an indication of how malleable it can be during special periods.

Previous studies on how children and adolescents use a mini version of the BarOn Emotional Quotient Inventory (EQ-i) test for youth (EQ-i: YV-Brief) indicated that TEI steadily enhanced with age (Keefer et al., 2013). Also, in a sample of adults with ages ranging from 19 to 22 years, Parker et al. (2005) reported moderate levels of 32-month test–retest associations for TEI (average *r* ¼ 0.55), implying that TEI is a rather malleable construct. Dave et al. (2021) used the EQ-i: Mini in a sample of adults, between ages 20–21 and 24–25, 4 years apart. Their results revealed that there was a moderate increase in TEI, suggesting the malleability of the construct during emerging adulthood.

In terms of measurement, to estimate TEI and its subdomains, Petrides (2009) introduced a scale called the Trait Emotional Intelligence Questionnaire (TEIQue). There are two versions of the questionnaire, namely, a long (TEIQue-LF) and a short (TEIQue-SF) form, which have been translated into several languages. These two versions offer the measurement of the global TEI and subscale scores on the four dimensions of the construct. These four dimensions are directly loaded on the global TEI, encompassing all four dimensions.

Alongside the ability EI and TEI, some psychologists have concentrated on the theory of constructed emotions (TCE), considering EI is at the center of the TCE model *via* its distinct attention to the emotional concept knowledge of individuals. Emotion concept knowledge (ECK) includes “modality-specific information about the facial muscle movements, vocal sounds, and bodily actions,” which are categories of different emotion words associated with a specific context (Doyle and Lindquist, 2017, p. 417). Barrett (2017a,b) conceptualized EI with regard to the concepts of emotion as understanding the feelings of others at a suitable time and occasion. She noted that individuals with high EI have a rich repertoire of emotional concepts, helping them build different emotional experiences (Barrett, 2017b). On the other hand, individuals with low EI may have limited emotion concepts in various emotional situations (Barrett, 2017b, p. 180). In contrast, individuals with high EI can construct emotion concepts, enabling them to associate each emotion concept with the appropriate affective context. Hence, they can expose, recognize, organize, and predict emotions “more efficiently” (Barrett, 2017b, p. 180). Moreover, Barrett (2017c) contended that expanding the ECK of an individual is more efficient for developing their EI.

Considering EI literature, this study focused on the trait model of EI. In other words, in this study, EI was viewed as a personality construct, placed at the lower levels of personality hierarchies (Dewaele et al., 2008; Alba-Juez and Pérez-González, 2019; Dewaele, 2019). This consideration was made for two reasons. Firstly, second or foreign language education has a communicative nature (Mercer and Gkonou, 2017), where learners interact and communicate with each other and with their teacher continuously. The construct of TEI, with its focus on both the inter- and intrapersonal EI (Li, 2020), is quite consistent with this communicative nature. Secondly, activities developed based on ability EI have been revealed to be demanding for learners, owing to the subjective nature of emotional experience. This shows that EI is “thus not amenable to truly objective scoring procedures” (Dewaele et al., 2008, p. 919). Instead, TEI seems more feasible in the language learning context.

### Trait Emotional Intelligence in Second Language Acquisition

The TEI exploration in Second Language Acquisition (SLA) is still in its fledgling state. For instance, Dewaele et al. (2008) found that language learners with high TEI had lower communicative anxiety in the L1 and lower levels of foreign language anxiety in their second, third, fourth, and fifth languages. They also maintained that high-TEI multilinguals indicated their potentiality to manage and integrate their feelings and ability to empathize with others. They also reported that high TEI helped learners to decrease their anxiety and evaluate whether their communication was satisfactory. On the other hand, low-TEI learners were revealed to have stayed in vague affective conditions while interacting with their interlocutors and fulfilling their communication with little potentiality to adopt if necessary, causing elevated anxiety.

Li and Xu (2019) investigated the association between two positive and negative emotions, i.e., FLE and foreign language classroom anxiety (FLCA), with TEI by adopting an exploratory sequential design. Their data were gathered from 1,718 Chinese senior high school learners by conducting a survey. Their results indicated the associations between TEI and both FLE and FLCA. Moreover, in the second qualitative phase of their study, 55 students answered open-ended questions after their participation in a 6-week positive psychology (PP)-inspired EI intervention. Their intervention consisted of the consciousness raising of learners and their reflection on their emotions. The findings of this study indicated the usefulness of such interventions in developing the positive emotions of students, decreasing negative ones, and enhancing their EI. More specifically, these results showed that students with high levels of TEI have high consciousness and control of their feelings when learning a language.

Li (2020) explored the association of TEI with emotions in learning experience (LX) learning. Her investigation of 1,307 Chinese high school LX learners of English indicated the positive association between TEI and achievements in FLE and LX. She also reported that learners with higher levels of TEI, who were going through higher levels of enjoyment in their achievement in language learning, achieved higher scores in their language achievement. Based on this finding, she argued that “other L2 classroom emotions may also […] co-mediate the relationship between TEI and L2 achievement” (p. 4).

Recently, Resnik and Dewaele (2020) focused on the crucial role of TEI in L1 and LX emotions in the language learning context. Their study on the positive and negative classroom emotions of 768 language learners in L1 (German) and LX (English) classes in the German-speaking world indicated a negative association between the anxiety ratings of language learners and their TEI. A positive correlation between the enjoyment experienced by the language learners in class and their TEI in both contexts was also found. Thus, these result showed that the link between anxiety and TEI was stronger than that of TEI and enjoyment, suggesting that anxiety should be regarded as more stable than enjoyment.

### Emotion Perception

Emotion perception is conceptualized as “inferring” the emotional context in other individuals using both verbal and non-verbal cues (such as facial expressions, tone, and body language) (Lindquist et al., 2016, p. 582). Thus, EP has been investigated from two different approaches, namely, the basic (traditional) emotion approach and the psychological constructionist approach. Traditionally, emotion expression and detection were assumed to be universal, automatic, and reflective (Ekman, 1972).

Specifically, the traditional or basic view of EP is in alignment with the group of basic emotions approaches (e.g., Izard, 2009; Shariff and Tracy, 2011). These approaches assume that all individuals, regardless of their culture, ethnicity, and individual differences, have a set of universal emotion categories (e.g., anger, hatred, joy, sadness, and fear) which are biologically, and not psychologically, generated in reaction to social and emotional stimuli (Ekman and Cordaro, 2011). According to these sets of approaches, emotions are assumed to be associated with facial expressions in a one-to-one manner; that is, the face universally and consistently generates distinct signs as individuals experience particular emotions (e.g., experiencing fear leads to widening eyes) (Tracy and Matsumoto, 2008).

On the other hand, constructionists proposed that both the expression and perception of emotions are not basically expressed and recognized universally. Instead, they are psychologically constructed through the use of ECK, which refers to the emotion categories that are used to comprehend the facial, vocal and verbal expressions of other individuals (Barrett, 2017b) in the mind of the expresser and perceiver of emotions (Barrett, 2017b; Doyle and Lindquist, 2017). According to a constructionist perspective, the universality aspect of ECK can be limited to valance (negative vs. positive) and arousal (low vs. high) (Russell, 1980; Russell and Barrett, 1999). The active role of the perceivers of emotions in building perceptions of their surrounding world according to expectations, individual differences, and category knowledge has been supported in this approach (Barrett et al., 2011; Hassin et al., 2013; Lindquist and Gendron, 2013). Thus, from the constructionist perspective, EP is perceiver dependent; that is, a perceiver has an active role in constructing and making meaning of the emotions of his or her interlocutor using his or her ECK (Barrett, 2017b).

Once the focus on the nature of EP shifted from a traditional perspective to a constructionist view, we noticed that there was no one-to-one association between a certain set of facial muscle movements and the recognition of an emotion. This might stem from the fact that EP is likely to vary across contexts, individuals, or cultures (Gendron and Barrett, 2017). Previous studies in the context of SLA have showed that the interpretation of emotion in bi or multilingual learners might be under the influence of several factors such as their TEI, proficiency level, context of language learning, and prior experience of language learning (Dromey et al., 2005; Dewaele et al., 2019; Alqarni and Dewaele, 2020). In the following sections, the influential role of TEI in language learners when perceiving the emotions of their interlocutors in the context of SLA is explained.

### The Link Between Trait Emotional Intelligence and Emotion Perception

As discussed previously, psychologists of TCE have investigated the association between TEI and EP with a focus on the role of the conceptual and lexical storage of individuals. Petrides and Furnham (2003) explored the effect of the TEI of psychology students on their ability to perceive emotional expressions, which are facial prototypical. Applying videos of female and male faces, they investigated the ability of students to perceive the facial prototypical emotional expressions of six basic emotions, namely, happiness, sadness, fear, anger, surprise, and disgust. They found that students with higher levels of TEI could recognize emotional facial expressions faster than those with lower levels of TEI.

Jacob et al. (2013) also investigated the association between EI and EP using a task-based EI estimation and an estimation of perceptual non-verbal dominance among 40 L1 users of German. Their results indicated that the recognition of their participants of the emotional state of the speaker was based on both verbal and non-verbal cues. Nonetheless, they found a stronger correlation between the overall mean valence estimations and those of the non-verbal channel. In contrast, at the individual level, the higher non-verbal dominance was associated with smaller reaction time differences between emotionally congruent and incongruent stimuli. Additionally, they pointed out that their participants with high levels of EI were more inspired by non-verbal cues when recognizing the target emotions. Thus, they suggested that the states of authentic emotions might be mainly communicated *via* non-verbal cues.

Following this avenue of research on the relationship between TEI and EP, Lea et al. (2018) conducted an eye-tracking experience with 54 UK adults. Their results revealed that high TEI was associated with tendencies for positive emotional stimuli. They also noted that this finding was in line with three of the facets of TEI, namely, well-being, self-control, and emotionality. Based on this finding, they argued that emotional scenes, but not isolated faces or images, are more socially noticeable and, hence, have more ecological validity.

Alqarni and Dewaele (2020) explored the EP of 333 English monolinguals, 205 Arabic-English bilinguals, and 71 Arabic monolinguals, and its relationship with the EI of these participants. They asked their participants to identify six basic emotions in 12 short video clips, six in English and six in Arabic. Their findings indicated that bilinguals performed better than English monolinguals in EP when the Arabic videos were played. They also found that bilinguals had higher TEI scores, with these scores being positively linked with their EP scores. With this result in mind, they noted that EI appears to be the one of the key psychological traits associated with EP.

Dewaele et al. (2019) also emphasized the interaction between TEI and language proficiency in EP. In their study, the participants were 150 British and 151 American L1 users of English watching some emotional stimuli. They found that language learners with lower proficiency depended more on their TEI for the interpretation of emotions than those with higher proficiency.

According to the above-mentioned studies, a link between TEI and EP can be created. However, this link has not been investigated in terms of TEI subdomains across time. A reflection on the previous studies regarding TEI in SLA prompts several unexplored areas. Currently, no research has yet explored the TEI as a longitudinal process in the field of the psychology of language learning. With this gap in mind, the current study intended to explore the co-development and interaction of its subdomains of TEL longitudinally. Moreover, considering the important role of TEI in EP, as mentioned in the previous studies (Dewaele et al., 2019; Alqarni and Dewaele, 2020; Mavrou and Dewaele, 2020), this study also aimed to incorporate EP as a distal outcome that could be influenced by the initial and growth level of the global factor of TEI and its subdomains.

### Factor-of-Curves Growth Curve Model

Currently, there exist only cross-sectional (Dewaele et al., 2019; Alqarni and Dewaele, 2020; Mavrou and Dewaele, 2020; Resnik and Dewaele, 2020) or pretest–posttest designs (Li, 2020) in studies that examine the TEI construct in the field of SLA. It should be noted that considering only two time points for exploring a continuous process provides only two discrete snapshots, which limits the understanding of the dynamic nature of psychological attributes (Karney and Bradbury, 1995). Researchers in different disciplines have criticized these conventional approaches for their limited capability to investigate and evaluate change in individual differences, including personal characteristics, behaviors, and education outcomes over time (Wickrama et al., 2016; Isiordia and Ferrer, 2018; Kiefer et al., 2018; Hiver and Al-Hoorie, 2020). Given the restrictions of traditional methodology, the latent growth curve model (LGCM) provides imperative methodological benefits for evaluating panel data over the traditional regression approaches and mean comparisons. This approach involves various advantages. Firstly, the capability of this model to evaluate the dynamic nature of different attributes can bring a more comprehensive understanding of individual differences as a continuous process over time, and not just as a status at two distinct time points (Coyne and Downey, 1991). Secondly, when individual change follows a non-linear trajectory, LGCMs are suitable to disclose the details of such trajectories (Willet and Sayer, 1994). Thirdly, LGCM approaches offer empirical researchers a broader range of research questions intertwined with the nature of individual development (Willet, 1988).

On the other hand, as the developmental patterns of individuals become more complex, different extensions of conventional LGCMs, such as second-order growth curve models with higher-order factor structures, have been introduced to appropriately explore fundamentally significant, yet multifaceted research questions (Wickrama et al., 2016). Many of these inquiries are likely to include multidimensional data structures with higher-order factor structures. Interestingly, a conventional LGCM does not permit for the evaluation of higher-order factor structures. Pointing out this limitation, a conventional LGCM can be extended to a second-order latent growth model to capture these potential higher-order factor structures in a longitudinal modeling.

With the FCM approach, the primary growth factors of specific subdomains are explicitly estimated. Thus, the primary growth factors and the covariations among these primary growth factors can be investigated. Therefore, for research questions aimed at investigating both primary growth factors and their covariations in an effort to realize the co-development of different attributes, the FCM approach is suitable (Wickrama et al., 2016).

Specifically, it is recommended that the FCM approach may be the most proper when exploring the co-development of time-varying constructs, such as TEI during a specific time period because it permits for the evaluation of both the trajectories of specific subdomains and the trajectories of the global second-order domain during stages or phases of rapid development (Wickrama et al., 2016); that is, risks that are specific to a given subdomain and shared risks across subdomains can be explored simultaneously, which makes this approach more applicable to rapid developmental periods when attributes change independently. However, this change may also permit the construction of second-order growth factors. Because primary growth factors specific to each subdomain are estimated *via* an FCM approach, the notion of the desired longitudinal covariance patterns of the repeated measures can be applicable to each of the primary growth curves.

In FCM, these first-order growth factors are extended to second-order global latent factors (Wickrama et al., 2016). Hence, the associations among these first-order growth factors of various subdomains ascertain the “loadings” of first-order growth factors as indicators of second-order global growth factors. The explained variance of each variable is defined by the square of the “loadings,” indicating the reliability of the first-order growth factors as indicators of the second-order growth factors (Wickrama et al., 2016). If acceptable loadings are found, an FCM is statistically suitable for exploring co-occurrence among various sub-factors.

An FCM can also be used to predict distal outcomes or consequences. Latent distal outcomes defined by single or multiple indicators can be predicted by growth parameters (e.g., intercept and slope of the global factor) and primary, time-specific, and latent variables (e.g., intercepts and slops of the first order factors). In this study, EP was used as a distal outcome to investigate how growth factors, including intercept and slope factors in a TEI-FCM, influence EP.

### The Current Study

**Figure 1:**
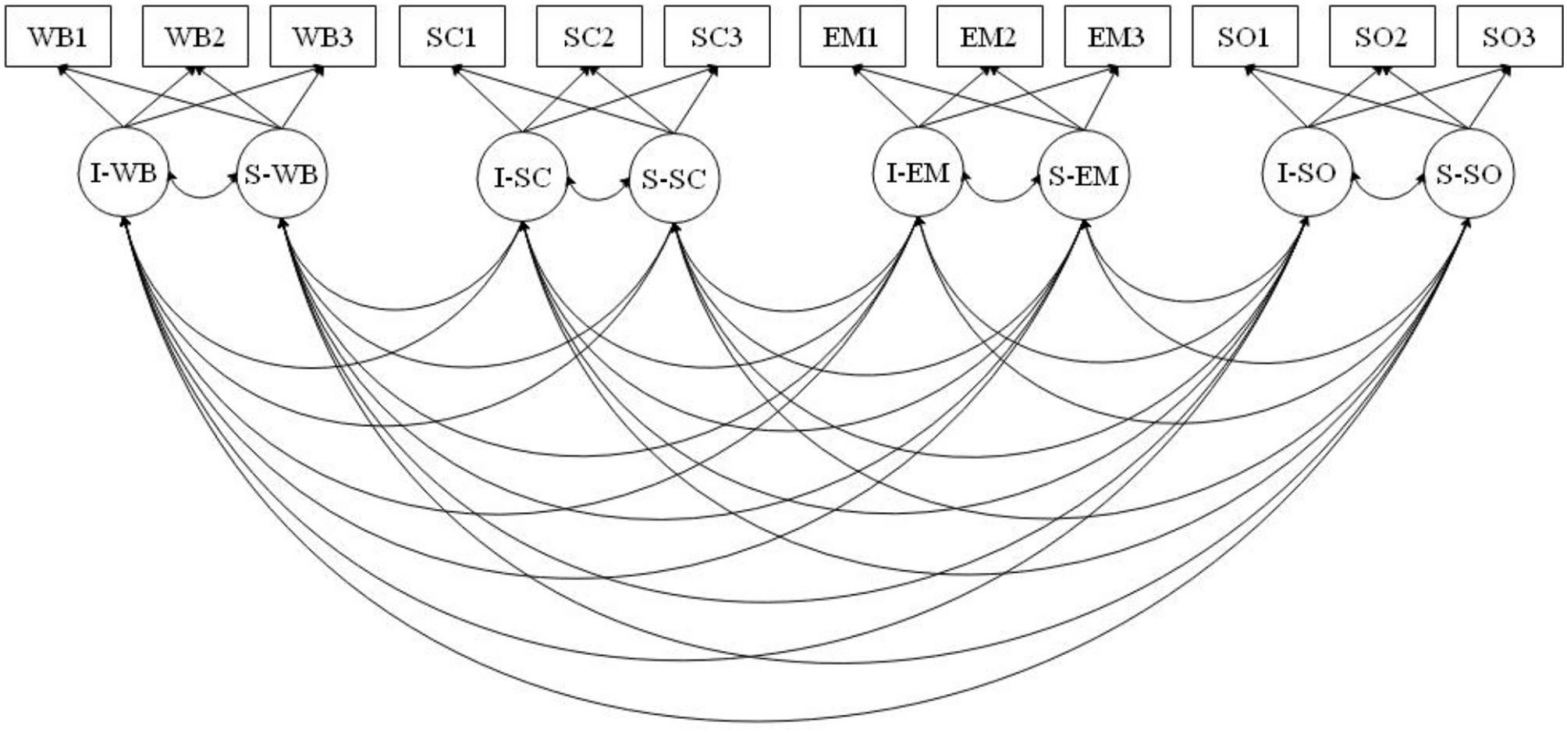
Parallel process model (PPM), a first-order longitudinal growth model (LGM). I, intercept; S, slope; TEI, trait emotional intelligence; WB, well-being; SC, self-control; EM, emotionality; SO, soociability.

**Figure 2:**
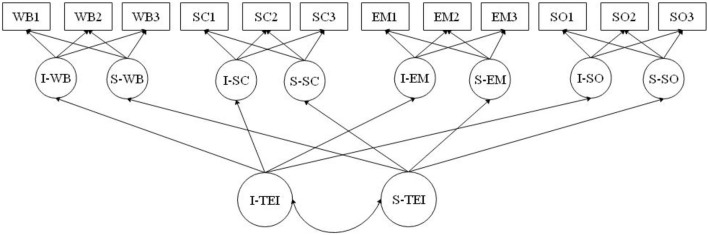
Factor-of-curves model (FCM), a second-order LGM. I, intercept; S, slope; TEI, trait emotional intelligence; WB, well-being; SC, self-control; EM, emotionality; SO, sociability.

Considering the advantages of the parallel process model (PPM) as a first-order longitudinal growth model (LGM) (Figure 1) and the FCM, which is an extended form of PPM as a second-order LGM (Figure 2), this study aimed to investigate both the primary growth factors of TEI and their covariations in order to examine the co-development of the different subdomains of TEI and the contribution of each subdomain to the global factor of TEI. A PPM has a set of first-order growth curves for correlated subdomains. *Via* the PPM, the association among primary growth factors can be tested to trace the co-development of different constructs and capture the communality in the primary growth factors. Additionally, since a PPM is viewed as a primer of a second-order growth curve (Wickrama et al., 2016), it can be extended to a second-order growth curve (FCM) to test to what extent the co-development of these subdomains is accounted by a global factor.

Additionally, using EP as a distal outcome, this study aimed to investigate how growth factors in a TEI-FCM influence the distal outcome of EP. Given these, four assumptions were considered for the present study. First, the primary, or first-order, growth factors (e.g., the initial level and slope) of a subdomain of TEI explain the developmental trajectory in that specific subdomain over time. Second, the covariations among growth factors of subdomains reflect a parallel process involving the four subdomains of TEI over time; that is, the slopes of each subdomain should run parallel to each other: as one increases, so do the others. Third, we assumed that the higher-order factors of TEI accurately reflect the variances and covariances among the primary growth factors. Finally, it was assumed that EP as a distal outcome could be predicted by the global growth parameters of TEI and one or more subdomains of TEI. Given these hypotheses, the following research questions were addressed:

1. To what extent and in what direction do TEI subdomains change over time?
2. To what extent are changes in well-being, emotionality, self-control, and sociability associated with their initial level?
3. To what extent are changes in well-being, emotionality, self-control, and sociability related over time?
4. To what extent does a second-order factor structure of the global domain of TEI describe the associations among the primary growth factors of its subdomains?
5. How does the global factor growth curve of TEI influence the EP of language learners?
6. To what extent do the growth parameters of the subdomains of TEI predict the EP of language learners?

## Methodology

### Participants and Setting

In the current study, convenience sampling approach was applied based on the access to language learners in the private institutes of four cities in Iran. The sampling setting included learners who were acquiring English as a foreign language (EFL) in these institutes. The data were collected from 28 classes with a range of 8–14 students per class. Initially, data were gathered from 336 learners (234 females, 102 males). However, 27 participants were removed from the study due to the lack of success in re-contacting the participants for a follow-up survey over the 1-year period of our data collection. Thus, the number of participants was reduced to 309 (217 females, 92 males). The first language of all the participants was Persian. They were all adult English language learners who were learning EFL. The data collection occurred from February 2020 to February 2021. Through a self-reported questionnaire, the participants provided information about their language proficiency in English, gender, age, nationality, their L1(s), and other languages. The proficiency level of this sample ranged between lower-intermediate and upper-intermediate. The proficiency levels of all the participants were also checked *via* an Oxford Placement Test. The average age of the participants was 28 years (SD = 10.4; range = 14–37 years) with a skewness of 0.58 (SE = 0.09) and a kurtosis of −0.21 (SE = 0.18). Their proficiency levels (*M* = 4.56 out of 5, *SD* = 0.71) resulted in a composition of beginner (13.4%), lower-intermediate (34.2%), intermediate (31.9%), upper-intermediate (19.1%), and advanced (1.4%) learners. Table 1 presents detailed information about the participants.

**Table 1:** Demographic information of participants.

**Institute**	**No**.	**Male**	**Female**.	**No. of classes**	**Mean age (SD)**
A	72	21	51	6	21.44 (8.01)
B	59	17	42	6	24.48 (7.49)
C	45	14	31	5	23.47 (5.11)
D	47	18	29	4	22.51 (4.98)
E	86	22	64	7	26.43 (7.54)
Total	309	92	217	28	23.66 (6.62)

### Instrumentation

The following instruments were used to collect the data in this study:

1. *The Trait Emotional Intelligence Questionnaire-Short Form (TEIQue-SF)* (Petrides, 2009).
2. *Emotion Perception Task*.

Firstly, the participants filled out the Persian-translated short version of the TEI Questionnaire (Petrides, 2009), with 30 items. The mean of the global TEI score was 4.12 (SD = 0.63), with scores ranging from 2.1 to 5.4 (absolute min = 1, absolute max = 7).

The TEI scale also allowed the estimation of scores on the four TEI subdomains: well-being, emotionality, self-control, and sociability. *Well-being* comprised items such as “I feel good about myself” and “I'm a very motivated person.” The mean score of this subdomain was 5.62 (SD = 0.98). *Self-control* was measured through items such as “I try to control my thoughts and not worry too much about things” and “I find it hard to keep myself motivated.” The mean score of this subdomain was 5.17 (SD = 0.91). *Emotionality* consisted of items such as “Sometimes, others complain that I treat them badly” and “It's easy for me to talk about my feelings to other people.” The mean score of this subdomain was 4.96 (SD = 0.64). *Sociability* included items such as “I'm unable to change the way other people feel,” “I am able to change the way other people feel,” “I find it hard to cope when things change in my life,” and “I find it easy to copy when things change in my life.” The mean score of this subdomain was 5.28 (SD = 0.82).

For this study, TEIQue-SF was translated into Persian and then translated back into English by two translators. Next, a comparison was done between the back-translated items and the original English items before the final Persian-translated version of the scale was finalized. Afterwards, the Persian version of the scale was piloted to 13 EFL learners, who were not among the participants of the study. They were asked to provide any vague and unclear items in terms of wording and structure. The items were presented to them in a random order. Based on their comments, modifications in some items were made. Finally, the Persian version of the TEIQue-SF was administered during regular classroom hours in three measurement occasions with 6-month time intervals. It took approximately 16 min to fill out the scale.

To check the reliability of the scale, Cronbach's α was used to assess the internal consistency and McDonald's ω (McDonald, 1985, 1999) to examine the composite reliability (Simşek and Noyan, 2013) of the questionnaire. The application of McDonald's ω and coefficient α was done due to “a tendency for coefficient α to underestimate the true reliability when the data are multidimensional” (Osburn, 2000, p. 343). Information about the reliability of the scale can be seen in Table 2.

**Table 2:** Reliability estimates of the TEI and its subdomains.

	**No of items in the final analysis**	**Cronbach's α (95% CI)**	**ω (95% CI)**
Well-being	6	0.83 (0.78–0.85)	0.81 (0.76–0.84)
Self-control	7	0.66 (0.68–0.73)	0.65 (0.62–0.68)
Emotionality	8	0.72 (0.69–0.75)	0.69 (0.67–0.72)
Sociability	8	0.70 (0.66–0.72)	0.68 (0.66–0.71)
Global TEI	30	0.78 (0.74–0.81)	0.77 (0.72–0.80)

The Emotion Perception Task (EPT) includes six short audiovisual clips representing examples of four negative emotions (anger, fear, sadness, and disgust) and two positive emotions (surprise and happiness). The videos were adopted from a study by Lorette and Dewaele (2019), which were characterized by daily conversations. In each video clip, an actress with a slightly British accent received pronunciation improvised a routine-life situation to transfer one of the six so-called “fundamental” emotions (happiness, sadness, surprise, disgust, anger, or fear). The reason for the selection of a female actress in these videos was that the emotions of females tend to be recognized more clearly than those of males (Lorette and Dewaele, 2019). Prompts to her emotional situation could be identified from both verbal and non-verbal cues (i.e., her facial expression and tone of voice).

### Data Collection

The participants were asked to answer several sets of questions. The first set involved questions on the semibiographical background of these participants, their age, their gender, their language learning history, and their self-perceived proficiency of these languages Additionally, the the Persian version of TEIQue-SF (Petrides, 2009) was given to the participants in three measurement occasions with 6-month intervals. Questionnaire completion was done step by step three times when one of the researchers was present. The participants were asked to write their student ID numbers so that we could easily match their ratings at three different occasions during the study. Finally, after distributing the scale in the third measurement occasion, the EPTs were presented to the participants, and they were asked to perform them according to instruction. Moreover, they were asked to write down their ID number for later correspondence. During the data collection, the participants were assured of the confidentiality of the information they provided and that they could leave the whenever they had difficulties in following the project.

### Data Analysis

The software program used to analyze the data in this study was Mplus 8.4 with a robust maximum likelihood estimator (MLR). The analysis followed the incremental steps for FCM recommended by Wickrama et al. (2016). The steps were, firstly, to check the possibility of forming primary growth curves for each subdomain of TEI, as the correlation patterns among indicators of these subdomains over time were investigated. To have a successful estimation of PPM, the correlation coefficients between two adjacent occasions (*t* and *t* + 1) should be higher than those at non-adjacent measurement occasions (Lorenz et al., 2004). Additionally, from each of the primary growth models, whether there was adequate inter-individual variation in the intercepts and slopes of the four subdomains of TEI was checked. It should be noted that significant variations in the intercepts and slopes of these subdomains provide empirical evidence for the possibility of forming a PPM and the estimation of the univariate primary LGCMs involving these subdomains. Secondly, auto-correlated measurement errors were also incorporated in the current PPM to reduce the chances of model misspecification that could introduce bias into the model parameter estimates (Wickrama et al., 2016). Thirdly, to obtain empirical evidence for the successful estimation of second-order growth factors for an FCM, the covariances among primary growth factors of the TEI subdomains, which were the indicators of the second-order growth curve, were examined (Wickrama et al., 2016). Finally, a conditional FCM incorporating time-invariant covariate of EP was conducted.

Furthermore, for testing the model fit, we applied the goodness of fit indices, such as the comparative fit index (CFI), Tucker-Lewis index (TLI), root mean square error of approximation (RMSEA), and standardized root mean square residual (SRMR). The acceptance criteria were CFI and TLI ≥0.9 and ≥0.95, and RMSEA and SRMR ≤ 0.08 and ≤ 0.05, pointing to adequate and excellent fit indices, respectively (Hu and Bentler, 1998; Marsh et al., 2004).

## Results

The results of analysis are presented here in four steps corresponding to the incremental steps of conducting an FCM procedure, based on which each research question was developed.

### The Longitudinal Correlation Patterns Among the Repeated Measures of Each Subdomain

As represented in Table 3, the correlation matrix reveals that the correlation coefficients between two adjacent occasions (*t* and *t* + 1) for each subdomain are higher than the correlations between non-adjacent occasions (well-being *rs* ranged from 0.4 to 0.48; self-control *rs* ranged from 0.28 to 0.38; emotionality *rs* ranged from 0.26 to 0.37; sociability *rs* ranged from 0.43 to 0.51). Moreover, off-diagonal correlations of the same repeated measure over time (in Table 3) are fairly different from each other. These correlations revealed that a significant slope variation might exist for each subdomain trajectory model.

**Table 3:** Correlation matrix between subdomains.

	**Well-being**			**Self-control**			**Emotionality**			**Sociability**		
	**WB1**	**WB2**	**WB3**	**SC1**	**SC2**	**SC3**	**EM1**	**EM2**	**EM3**	**SO1**	**SO2**	**SO3**
WB1	–											
WB2	0.48	–										
WB3	0.40	0.46	–									
SC1	0.71	0.41	0.35	–								
SC2	0.39	0.73	0.37	0.38	–							
SC3	0.28	0.34	0.75	0.28	0.37	–						
EM1	0.65	0.29	0.23	0.70	0.31	0.28	–					
EM2	0.36	0.67	0.27	0.23	0.66	0.21	0.34	–				
EM3	0.29	0.24	0.64	0.22	0.19	0.68	0.26	0.37	–			
SO1	0.71	0.32	0.31	0.73	0.34	0.38	0.69	0.41	0.39	–		
SO2	0.43	0.66	0.37	0.31	0.68	0.30	0.41	0.64	0.40	0.47	–	
SO3	0.39	0.34	0.72	0.46	0.44	0.70	0.39	0.338	0.71	0.43	0.51	–

*WB, well-being; SC, self-control; EM, emotionality; SO, sociability*.

### Parallel Process Growth Curve Model

As seen in Table 4, the intercept and slope variance of each model are also correlated. The model results showed that all between-subdomain auto-correlated errors were statistically significant and within the acceptable bounds, ranging from 0.68 (PE1 with CI1) to 0.76 (PE4 with CI4) (Table 4). Additionally, the model fit was acceptable [$\chi_{\left(\text{df}\right)}^{2}$ = 211.127 (98), *p* < 0.001; CFI/TLI = 0.951/0.947; RMSEA = 0.041; SRMR = 0.052].

**Table 4:** Results of the parallel process model (PPM).

	**Intercept growth factors**		**Slope growth factors**			**Correlations among growth factors**							
	**M**	**V**	**M**	**V**		**I-WB**	**I-SC**	**I-EM**	**I-SO**	**S-WB**	**S-SC**	**S-EM**	**S-SO**
WB	5.62^***^	0.231^***^	0.59^***^	0.011^***^	I-WB	–							
					I-SC	0.712^***^	–						
SC	5.17^***^	0.198^***^	0.29^***^	0.013^***^	I-EM	0.769^***^	0.735^***^	–					
					I-SO	0.754^***^	0.722^***^	0.706^***^	–				
EM	5.01^***^	0.237^***^	0.21^***^	0.009^***^	S-WB	0.603^***^	0.528^***^	0.512^***^	0.451^***^	–			
					S-SC	0.574^***^	0.637^***^	0.537^***^	0.514^***^	0.854^***^	–		
SO	5.17^***^	0.246^***^	0.33^***^	0.007^***^	S-EM	0.484^***^	0.517^***^	0.613^***^	0.498^***^	0.819^***^	0.843^***^	–	
					S-SO	0.391^***^	0.493^***^	0.448^***^	0.581^***^	0.864^***^	0.858^***^	0.794^***^	–

*WB, well-being; SC, self-control; EM, emotionality; SO, sociability; I, intercept; S, slope; M, mean; V, variance [$\chi_{\left(df\right)}^{2}$ = 211.127 (98), p < 0.001; CFI/TLI = 0.951/0.947; RMSEA = 0.041; SRMR = 0.052]*,

*** p < 0.001; **p < 0.01.

The PPM findings indicated the existence of both between- and within-subdomain auto-correlated error structures (Table 4). With regard to the first research question, i.e., the direction and amount of change in the four subdomains of TEI, the PPM results showed a statically significant increase in the well-being (*M*_*Intercept*_ = 5.62, *SE* = 0.13, *p* < 0.001, *M*_*Slope*_= 0.89, *SE* = 0.19, *p* < 0.001), self-control (*M*_*Intercept*_ = 5.17, *SE* = 0.17, *p* < 0.001, *M*_*Slope*_ = 0.29, *SE* = 0.21, *p* < 0.001), emotionality (*M*_Intercept_ = 5.01, *SE* = 0.11, *p* < 0.001, *M*_Slope_ = 0.21, *SE* = 0.09, *p* < 0.001), and sociability (*M*_*Intercept*_ = 5.17, *SE* = 0.18, *p* < 0.001, *M*_*Slope*_ = 0.33, *SE* = 0.23, *p* < 0.001) of participants over the 1-year period. Moreover, the values of the intercept variance were 0.231 (*p* < 0.001) for well-being, 0.198 (*p* < 0.001) for self-control, 0.237 (*p* < 0.001) for emotionality, and 0.246 (*p* < 0.001) for sociability, suggesting that the initial levels of the four subdomains of TEI varied significantly among individuals. In addition, the variances of the slopes were for 0.011 (*p* < 0.001) for well-being, 0.013 (*p* < 0.001) for self-control, 0.009 (*p* < 0.001) for emotionality, and 0.007 (*p* < 0.001) for sociability. This also confirms that there were sufficient inter-individual variation and intra-individual trends within each subdomain and a significant increase over time in the four subdomains.

Moreover, all the covariances among the primary growth factors were also statistically significant, which implied the existence of a parallel process of growth across the subdomains of TEI. It is noteworthy that the correlations (standardized covariances) *among* the intercepts of these subdomains were higher than those *between* the intercepts and slopes of each subdomain (Table 4). In the same vein, correlations *among* the slopes of subdomains were higher than the correlations *between* the intercepts and slopes. These relatively strong correlations among the same type of growth parameters across the four subdomains point to the existence of significant global (second-order) growth factors in an FCM.

### Estimating an Unconditional FCM

To achieve empirical proof of the successful estimation of second-order growth factors for an FCM of TEI subdomains, the covariances or correlations among primary growth factors of these subdomains, as the indicators of the second-order growth curve, were supposed to be checked first. Regarding the second research question, the association of the initial level of each subdomain with its slope, the PPM results showed moderately high correlations between the intercept and slope growth factors within these subdomains (Table 4); that is, the correlation between the intercept and slope factors were 0.603, *p* < 0.001 for well-being, 0.637, *p* < 0.00 for self-control, 0.613, *p* < 0.001 for emotionality, and 0.581, *p* < 0.001 for sociability (Table 4). Given the third research question, which addressed the association between the changes in the four subdomains of TEI, as seen in the estimated PPM of Table 4, the covariances, presented in their standardized form as correlations, between the intercept growth factors turned out to be moderately high (ranged from 0.769 to 0.854*, p* < 0.001). Furthermore, the correlations between the slope factors were also high (ranged from 0.743 to 0.864, *p* < 0.001). These findings point to the existence of significant second-order growth factors. These moderately high correlations may show the need to incorporate correlations between the intercept and slope factors of each of the primary growth models in an FCM. In this FCM, the loadings and means of the well-being trajectories were fixed to 1 and 0, respectively, to estimate the means of the second-order intercept and slope factors using the scale of well-being (i.e., marker variable approach).

Here, Table 5 shows that the factor loadings for all the primary growth factors on the global factors were high and statistically significant, which means that each of the primary growth factors contributed significantly to defining the global factors. The mean and variance of the global intercept were 5.216 and 0.192, respectively. In addition, the mean and variance of the global slope factor were 5.632 and 0.004, respectively. All these global parameters were statistically significant at the *p* < 0.001 level. Regarding the fourth research question, the R-square statistics for each growth factor indicated that the global intercept factor accounted for approximately 90.1% of the variation in the primary intercept factors for well-being, 61.4% of the variation for self-control, 76.4% of the variation for emotionality, and 79.4% of the variation for sociability. Furthermore, approximately 91.2, 59.8, 72.6, and 75.8% of the variations in the primary slope factors for well-being, self-control, emotionality, and sociability, respectively, were accounted for by the global TEI slope factor. The positive TEI slope factor also showed an overall increase in global TEI over time. The model results also showed that the growth parameters (i.e., means and variances) of the global intercept and slope factors (I-TEI and S-TEI) were statistically significant, suggesting that significant inter-individual variations existed for the second-order intercept and slope factor means.

**Table 5:** Parameter estimates for FCM.

	**Estimates**	**S.E**.	**Est./S.E**.	**Two tailed *P*-value**
I-TEI by I-WB	1.000	0.000	999.000	999.000
I-TEI by I-SC	0.780	0.051	15.278	0.000
I-TEI by I-EM	0.816	0.049	17.123	0.000
I-TEI by I-SO	0.851	0.052	15.582	0.000
S-TEI by S-WB	1.000	0.000	999.000	999.000
S-TEI by S-SC	0.780	0.058	15.278	0.000
S-TEI by S-EM	0.816	0.049	17.123	0.000
S-TEI by S-SO	0.851	0.052	15.582	0.000
I-WB with S-WB	0.008	0.008	6.200	0.012
I-SC with S-SC	0.047	0.006	2.333	0.018
I-EM with S-EM	0.173	0.007	2.428	0.211
I-SO with S-SO	0.121	0.005	2.400	0.198
I-TEI with S-TEI	0.238	0.008	2.625	0.000
**Means**				
I-TEI	5.216	0.054	9.659	0.000
S-TEI	0.632	0.048	11.712	0.001
**Variances**				
I- TEI	0.192	0.034	6.205	0.000
S- TEI	0.004	0.003	1.667	0.000
**Residual variances**				
I-WB	0.052	0.021	2.468	0.000
S-WB	0.002	0.014	0.142	0.001
I-SC	0.019	0.004	4.68	0.032
S-SC	0.000	0.000	1.211	0.041
I-EM	0.048	0.017	2.841	0.005
S-EM	0.013	0.008	1.632	0.000
I-SO	0.027	0.021	1.284	0.139
S-SO	0.009	0.006	1.500	0.127
**R-square**				
I-WB	0.901	0.042	18.184	0.000
S-WB	0.912.	0.048	15.132	0.000
I-SC	0.614	0.059	10.384	0.000
S-SC	0.598	0.019	31.456	0.000
I-EM	0.764	0.048	18.741	0.000
S-EM	0.726	0.057	16.317	0.000
I-SO	0.794	0.072	11.027	0.000
S-SO	0.758	0.074	10.246	0.000

### Estimating a Conditional FCM: EP as a Distal Outcome

**Figure 3:**
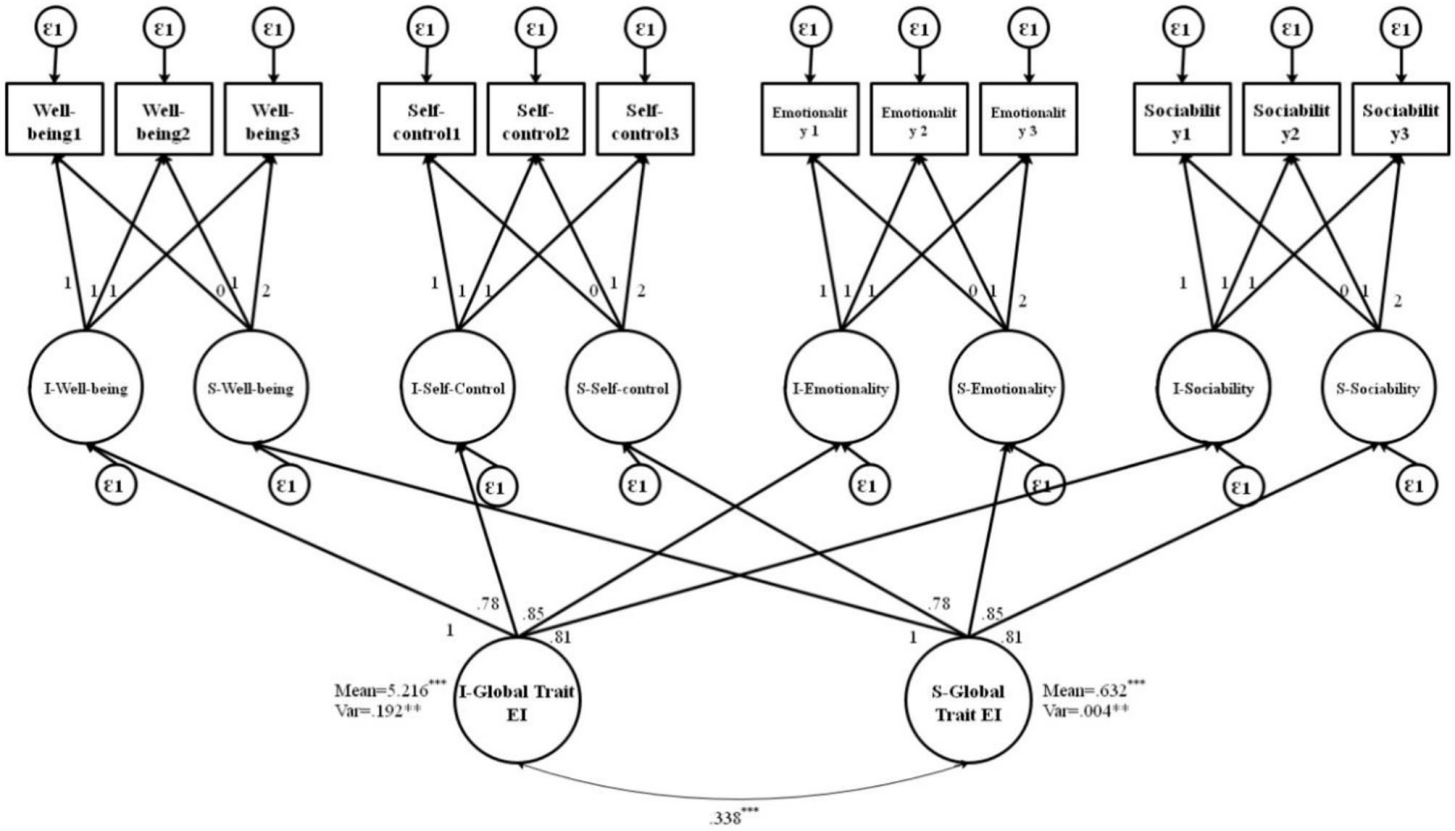
Factor-of-curves model (FCM) of TEI. I, intercept; S, slope; TEI, trait emotional intelligence; WB, well-being; SC, self-control; EM, emotionality; SO, sociability; M, mean; Var, variance; ε = error; γ = factor loading [$\chi_{\left(\text{df}\right)}^{2}$ = 236.84 (112), *p* < 0.001; CFI/TLI = 0.962/0.951; RMSEA = 0.051; SRMR = 0.055]; ***p < 0.001; **p < 0.01.

**Figure 4:**
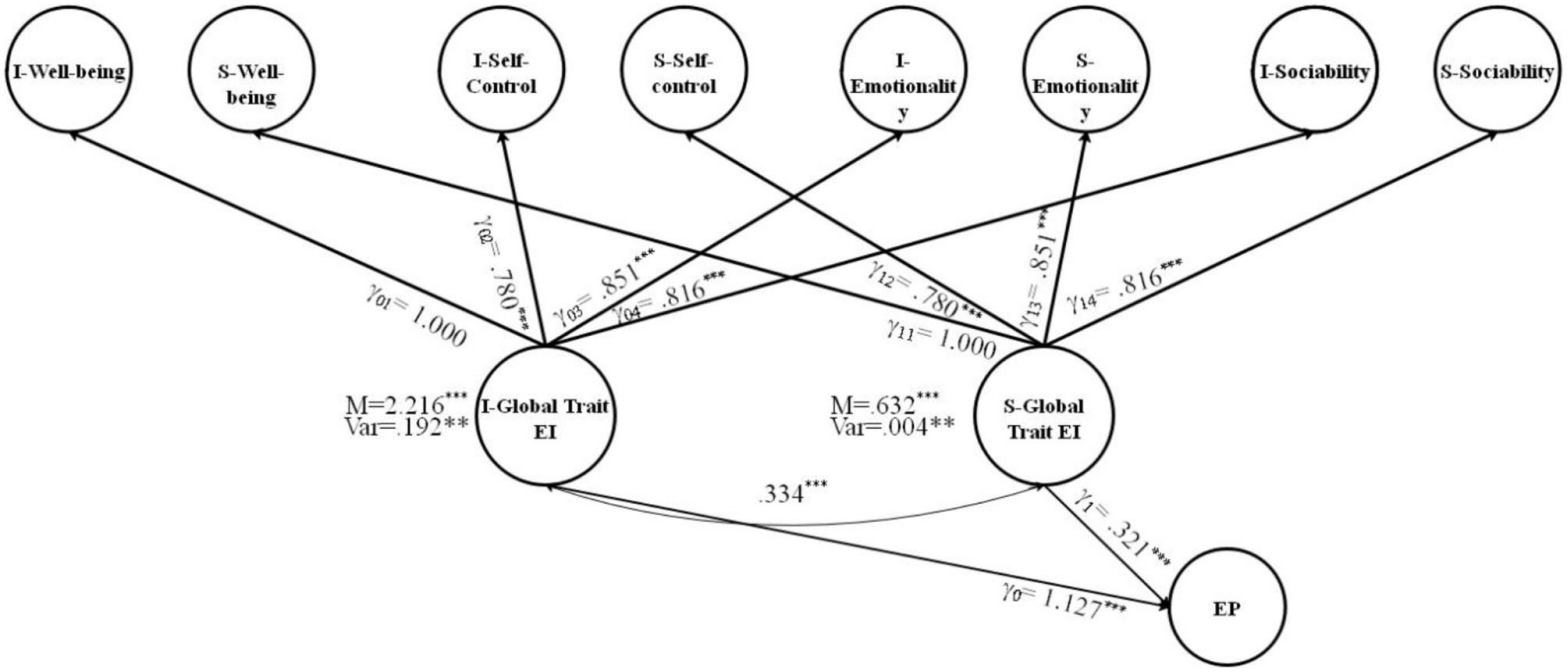
FCM of TEI with the a latent predictor variable (EP). I, intercept; S, slope; TEI, trait emotional intelligence; WB, well-being; SC, self-control; EM, emotionality; SO, sociability; EP, emotion perception; M, mean; Var, variance; ε = error; γ = factor loading [$\chi_{\left(\text{df}\right)}^{2}$ = 244.128 (98), *p* < 0.001; CFI/TLI = 0.974/0.963; RMSEA = 0.052; SRMR = 0.057]; ***p < 0.001; **p < 0.01.

**Figure 5:**
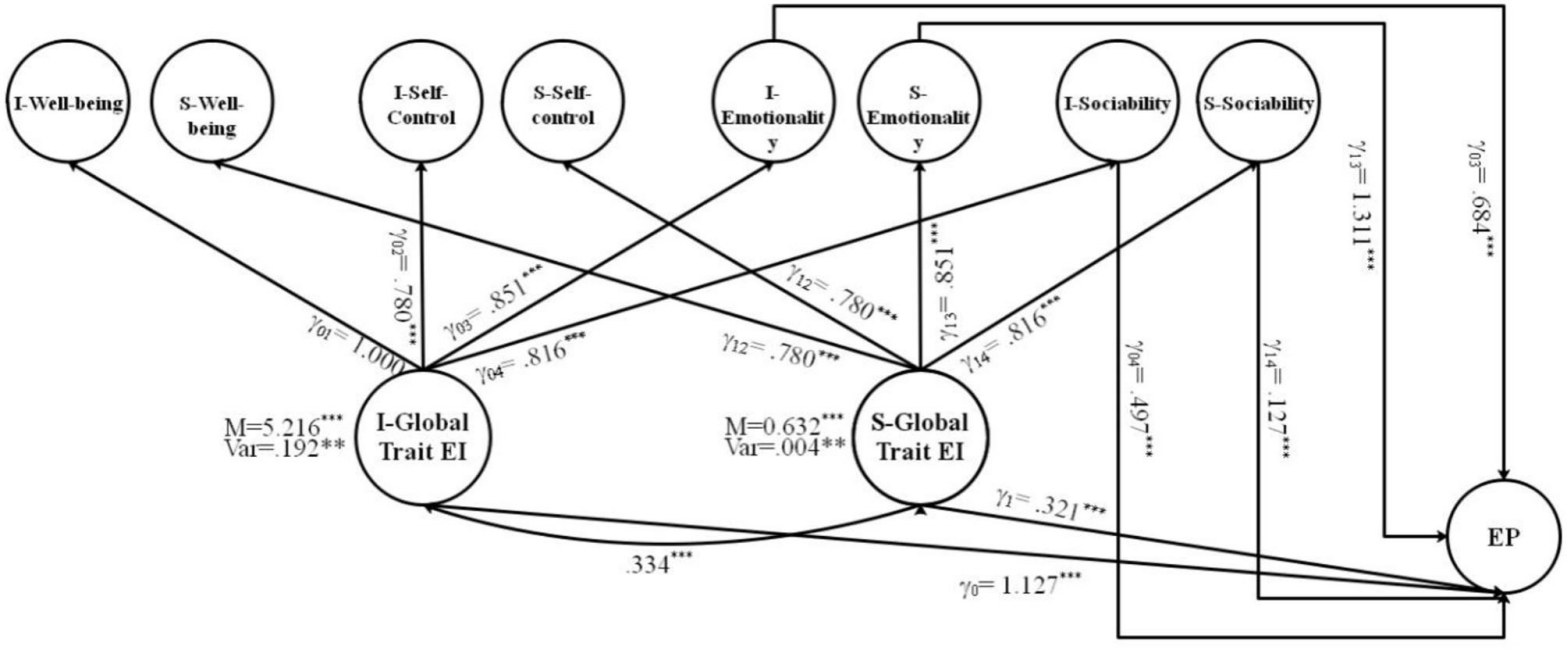
A full FCM of TEI with the a latent predictor variable (EP), incorporating significant paths from EP to the subdomains. I, intercept; S, slope; TEI, trait emotional intelligence; WB, well-being; SC, self-control; EM, emotionality; SO, sociability; EP, emotion perception; M, mean; Var, variance; ε = error; γ = factor loading [$\chi_{\left(\text{df}\right)}^{2}$ = 261.614 (97), *p* < 0.001; CFI/TLI = 0.982/0.973; RMSEA = 0.053; SRMR = 0.057]; ***p < 0.001; **p < 0.01.

The current study also estimated the direct and indirect effects of TEI and its subdomains on a latent predictor, EP, by specifying paths from both subdomain-specific latent factors and second-order growth factors. The FCM with EP as an exogenous latent variable is shown in **Figures 4**, **5**. Figure 3 corresponds with predicting the potential of global growth factors of TEI for predicting EP, while Figure 3 corresponds to predicting the potential of both primary and global growth factors of TEI for predicting EP. The covariance was constrained between the global growth factors to be zero for model identification purposes in Figure 3. The results are shown in Figures 4, 5.

To answer the fifth research question, regarding the direct effects of the global growth factors (intercept and slope of TEI) on the distal outcome (i.e., EP) after controlling for the effects of the primary growth factors, the results indicated that the intercept and slope of global TEI were associated with EP. The direct effect of the initial level of TEI on EP (γ_0_ = 1.127, *p* < 0.001) and the direct effect of the growth of TEI on the EP *(*γ_1_ = 0.321, *p* < 0.001) are interpreted as a 1.127 increase in the TEI intercept and a 0.321 increase in the TEI slope for each one unit increase in EP after controlling for the influence of growth factors (Figure 4).

With regard to the sixth research question, in the full FCM (Figure 5), the effects of a primary growth factors (i.e., the intercepts and slopes of well-being, self-control, emotionality, sociability) on the distal outcome of EP were estimated after controlling for the effects of the global growth factors. The results indicated that TEI secondary intercept and slope factors not only directly influenced the EP, but also indirectly influenced it through the intercept and slope factors from two primary growth models (emotionality and sociability); that is, the increase in emotionality and sociability was associated with the increase in EP over time after controlling for the effects of the global domain (TEI) EP (γ_03_ = 1.311, *p* < 0.001; γ_13_ = 0.684, *p* < 0.001; γ_04_ = 0.497, *p* < 0.001; γ_14_ = 0.127, *p* < 0.001). There was no significant path from the intercepts and slopes of the well-being and self-control to EP.

## Discussion

The current study investigated the trajectories of the global factor of TEI and the parallel development of the subdomains of TEI (e.g., well-being, emotionality, self-control, and sociability) using PPM and FCM over 1 year in the context of a foreign language classroom. Additionally, EP was used as a distal outcome in the model of the study to investigate how the growth parameters (including intercept and slope factors) in a TEI-FCM influence the distal outcome of EP. The following sections contain a discussion of the answers for the six research questions addressed in this research. It is important to point out that the previous studies on TEI did not investigate this construct longitudinally. Using FCM as an extended version of LGCM, we aimed to explore a richer and broader spectrum of the dynamic interaction of TEI subdomains and their co-development.

With regard to the first research question (To what extent and in what direction do TEI's subdomains change over time?), addressing the direction and amount of change in four subdomains of TEI (e.g., well-being, emotionality, self-control, and sociability), the PPM results showed a statically significant increase over 1 year in these subdomains. Pekaar et al. (2020) argued that the growth of the TEI and its subdomains can be influenced by some factors, such as situational cues, which elicit emotions in others and oneself and contextual and dispositional factors that might qualify the process of TEI dynamicity. With this argument in mind, it can be conjectured that both the situational cues and the contextual factors during a foreign language course might have contributed to the increase in the subdomains of TEI of students.

In addition, the intercept and slope variances for the four subdomains were statistically significant, which implies that there was adequate inter-individual variation within each subdomain. This significant variance in the initial level and growth of each of these subdomains indicated that some L2 learners had a higher rate of change in one or more subdomains of TEI compared with other learners with a lower rate of variation in the subdomains over time, while other learners preserved the same level of well-being, emotionality, self-control, and sociability over time. A related argument to this finding is the ergodicity issue regarding language students. Despite the increasing pattern in the four subdomains of TEI on average, this increasing pattern did not reflect changes in these subdomains at the individual level. This is due to the fact that language learners are not ergodic ensembles (Lowie and Verspoor, 2019) as the statistic on the average level does not necessarily provide the same results at the individual level (Tarko, 2005). Consequently, the ergodicity issue in research on individual differences suggests that the mean score of the attributes of individual learners is not essentially illustrative of the reality of the target attributes. There are cases of individual variation that are covert in the mean-based measurement of group attributes or behaviors. Thus, further case studies could clarify the nuances of variation in well-being, emotionality, self-control, and sociability. The existing TEI literature suggests a range of individual differences that may contribute to the significance of variance in the intercept and slope of the TEI subdomains. These individual differences can be age (Doerwald et al., 2016), cognitive intelligence (Côté and Miners, 2006), personality (Van der Linden et al., 2017), and gender (Fischer et al., 2018).

As for the second research question (To what extent are changes in well-being, emotionality, self-control, and sociability associated with their initial levels?), the association of the initial level of each subdomain of TEI with its slope, the PPM results revealed moderately high and positive associations between intercept and slope growth factors within the subdomains of TEI. These moderately high correlations within the subdomains showed that learners who started off with a higher level of well-being, emotionality, self-control, and sociability had more growth in these subdomains over time, while those who started off with a lower level of each subdomain had less growth over the term. This finding can be discussed in several ways, yet it most importantly points to the fact that the rate of change in TEI over time is susceptible to its initial states. This provides evidence for the dynamic nature of TEI, as changes in the initial states of a dynamic system influence the subsequent states of the system over time (de Bot et al., 2007). The growth susceptibility of each TEI subdomain to its initial level can be further explained in terms of spillover effects (Hareli and Rafaeli, 2008; Bakker and Demerouti, 2013). Spillover effects imply that the emotion of an individual at the end of an emotional episode influences his or her emotional experiences in the next episodes. Thus, with a focus on time, which is a neglected parameter in EI literature (Mesquita and Boiger, 2014), the positive association between the intercepts and slopes of TEI subdomains highlights the role of spillover in the dynamics of these subdomains over time. This also indicates why an analytic approach like the FCM is required to explore well-being, emotionality, self-control, and sociability of language learners over time and not just on one occasion of measurement.

Regarding the third research question (To what extent are changes in well-being, emotionality, self-control, and sociability related over time?), the PPM results indicated that that the covariances among first-order growth variables (e.g., well-being, emotionality, self-control, and sociability) were positive and statistically significant. This confirms the existence of a parallel process of growth across the subdomains of TEI. These non-directional correlations between and within the sub-domains of TEI point to the co-occurrence and co-development of subdomains of TEI over time. Thus, it could be concluded that well-being, emotionality, self-control, and sociability are the sub-domains of a single global factor (TEI), and the significant positive covariances among the intercepts and the slopes of these subdomains suggest that they construct a unique underlying latent factor called TEI. As the PPM findings indicated, these covariances are moderate. If, otherwise, they were high, the four sub-domains could be regarded as one subdomain. If they were rather low, the internal validity of the TEI would be questioned. Still, the moderate correlations prove the construct validity of the latent variable of TEI and its subdomains, which are adequately independent and at the same time positively associated. Another argument worth notifying about in this third result was that the covariance of the slopes was higher than that of the intercepts. This result indicates that, despite the variation in the initial levels of TEI subdomains, the rate of growth in the four subdomains enjoyed a stronger association.

Additionally, the significant and positive covariances among the intercepts and slopes of the subdomains confirm that a dynamic process exists between the growth models of the four dimensions of TEI. In other words, an increase in the early level of one subdomain affects the future growth rate of the other. Learners who consider themselves emotionally efficient (Emotionality) also tend to perceive themselves as socially efficient (Sociability), more determined (Self-control), and more adaptable in general (Well-being) over time. Such unidirectional, longitudinal impacts indicate a cumulative effect over time (Wickrama et al., 2016); that is, the effect of one subdomain on the other subdomain is reinforced over time, which leads to the co-development of the four subdomains over time. This result is consistent with the literature of TEI showing that the four dimensions of TEI are inter-correlated and work hand in hand to form a cohesive construct of TEI (Petrides, 2009; Dewaele, 2019). This cumulative effect can be postulated to be influenced by individual and/or contextual factors such as motivation (Pekaar et al., 2020), positive and negative emotions (Li and Xu, 2019; Li et al., 2021), and the relationship between the self and the other (Pekaar et al., 2020).

Furthermore, regarding the fourth research question (To what extent does a second-order factor structure of the global domain of TEI describe the associations among the primary growth factors of its subdomains?), the FCM results showed that the factor loadings for the four primary growth factors on the global factors were high and statistically significant, which indicates that each of the primary growth factors contributed significantly to defining the global factor of TEI. The results also indicated that the primary growth factors of the four dimensions of TEI differentially contribute to the second-order growth factors of TEI. More specifically, higher R-square values of well-being and sociability point to the heavier influence of these two subdomains of TEI on the global factor in constructing the context of language learning when compared to emotionality and self-control.

The important role of well-being in boosting TEI might not be surprising since learners who are positive and confident are better at dealing with the challenges and difficulties of learning a new language (Petrides, 2017; Dewaele, 2019). They integrate their enjoyment and satisfaction with learning a new language and are optimistic and confident about their progress in learning the language (Cuéllar and Oxford, 2018). The salience of sociability is equally clear, as learning a new language is essentially a social activity. Learners with a high level of sociability enjoy social interactions, are able to affect the feelings their interlocutors, and possess the required social skills to build friendly bonds with their classmates (MacIntyre et al., 2016). They are also good conversationalists and can interact confidently with interlocutors from different backgrounds.

However, the role of the two other TEI dimensions, emotionality and self-control, should not be ignored. Learners who have a high level of emotionality have an advantage in developing the required skills for communicating with their interlocutors (Petrides, 2017). Specifically, their empathic skills help them take the perspectives of others and perceive their emotions. Moreover, the emotionality dimension enables them to express their feelings well and build a good rapport with others, which could help them to be good language learners (Dewaele, 2019). Finally, learners with high self-control can regulate their own emotions, resist impulsive reactions, and manage stressful conditions (Chan, 2006; Brackett et al., 2010; de Costa et al., 2018).

To answer the fifth research question regarding the direct effects of the global growth factors (intercept and slope of TEI) on the distal outcome (i.e., EP) after controlling for the effects of the primary growth factors, the results indicated that the intercept and slope of global TEI were associated with EP (γ_0_ = 1.127, *p* < 0.001; γ_1_ = 0.321, *p* < 0.001). This complements the findings of previous studies, showing that high-TEI learners are better in perceiving and identifying the emotions of others in the context of SLA (Dewaele et al., 2019; Alqarni and Dewaele, 2020; Mavrou and Dewaele, 2020). This finding implies that individuals with high TEI can identify consensual emotions across different domains as they can notice the thought processes of others.

The possible mechanisms underlying the intercept and growth of global TEI with EP could be explained by the TCE (Barrett, 2017a,b). According to this theory, EI is “about getting your brain to construct the most useful instance of the most useful emotion concept in a given situation” (Barrett, 2017b, p. 179). Thus, learners with high TEI are interpreted to have a large repertoire of rich emotional concept knowledge, i.e., knowledge related to modality-specific information with respect to movements in the facial muscle, vocal sounds, kinetic actions, and categories of different emotion words associated with a specific context (Doyle and Lindquist, 2017). This emotional concept knowledge allows them to construct emotional experiences with subtle shades and differences and help them to recognize the emotions of others more accurately. Also, for someone who exhibits low TEI, this emotional concept knowledge might be limited, making the process of identifying the emotions of others more difficult; that is, the mind of an emotionally intelligent learner is better prepared to construct more emotion concepts, subsequently allowing him or her to associate each emotion word with the most proper emotional situation. Thus, they can perceive emotions “more efficiently” by experiencing, predicting, and categorizing them (Barrett, 2017b, p. 180).

With regard to the sixth research question (To what extent do the growth parameters of the subdomains of TEI predict the EP of language learners?), in the full FCM (Figure 5), the intercept and slope factors of only two subdomains of TEI, emotionality and sociability, turned out to be significantly linked to EP; that is, any increase in emotionality and sociability over time was associated with an increase in EP. This finding seems meaningful, as higher emotionality levels could be an indication of the better ability of learners to perceive the emotions of others. Thus, if some individuals are better than others at detecting the emotions of others, it might be due to their higher levels of emotionality and sociability. As an explanation for this finding, we can note that, since both emotionality and sociability are two other-focused dimensions of TEI, they seem to play a more active role in the regulation of the emotions of others compared with well-being and self-control, which reflect the self-dimensions of TEI. Rooted in the theories of social competence (Rose-Krasnor, 1997) and social-information processing (Crick and Dodge, 1994; Lemerise and Arsenio, 2000), the other-focused dimensions of TEI contribute to the manipulation of the psychological states of other individuals *via* encoding social cues in different social situations and, therefore, helping language learners to perceive the emotions and mood states of others better than the self-focused dimensions of TEI.

The overall findings of this research showed that the FCM procedure was a privileged and comprehensive analytical approach for the exploration of the co-development of the well-being, self-control, emotionality, and sociability subdomains of the TEI of L2 learners in the dynamic context of a language class. This statistical procedure proved effective in unraveling inter-individual variations and the results of testing the effect of the global latent variable on the initial state and intercept of the sub-domains of TEI as they naturally developed in the temporal phases of a language course.

## Conclusion

Given the dynamic turn in SLA, more dynamic process-based approaches have drawn the attention of researchers in this field, enabling them to trace the dynamics of their constructs of interest. Such approaches have already contributed to the understanding of anxiety and enjoyment dynamics in SLA (Dewaele and Dewaele, 2017, 2020; Elahi Shirvan and Taherian, 2018; Elahi Shirvan et al., 2021) and those of motivation (Hiver and Larsen-Freeman, 2020; Papi and Hiver, 2020), paving the way for the exploration of the dynamics of some other psychological constructs in SLA such as TEI. With this in mind, the study intended to examine the dynamics and temporal changes of TEI *via* the application of an innovative CDST-compatible method, which enabled the exploration of the different dynamic aspects of the TEI subdomains. First, the interplay of the different subdomains of TEI and their weight of contribution to the global construct of TEI can be investigated. Secondly, the multilevel format of the model of the study enabled the examination of individual differences in TEI, thus characterizing the dynamic perspective of TEI. Third, the TEI-FCM also incorporated the TEI subdomains in the exploration of TEI dynamics. Finally, the model of the study also incorporated EI as a distal outcome of TEI. The inclusion of EP in investigating the developmental process of TEI over time could shed light on the interplay between different TEI dimensions when individuals are processing the motions of others.

The findings indicated a statistically significant increase in the TEI subdomains over 1 year. Furthermore, the covariances among the first-order growth variables of these subdomains were revealed to be positive and statistically significant. Moreover, the findings indicated that the subdomains of TEI contributed significantly to the temporal changes of TEI. Furthermore, the results indicated a significant association between the initial and growth scores of TEI with EP. Specifically, only two subdomains of TEI, emotionality and sociability, turned out to be significantly linked to the EP. Regarding the pedagogical implication of these findings, it can be noted that language teachers should not limit their evaluation of the TEI their students to a specific session in an EFL course. Rather, they should regard it as a dynamic state that changes over time. This means that they should trace the factors that can enhance the TEI of their learners, while still taking their individual differences into account. Furthermore, they should view TEI as a multidimensional construct in which each dimension of TEI contributes differently to the changes of their learners in this construct. Furthermore, teachers should keep a long-term eye on the association between the EP and TEI of their learners; that is, the efforts of teachers to increase the TEI of their learners during an EFL course might strengthen their EP and even the emotional scaffolding of teachers. Lastly, concerning the limitations of the study, the incorporation of a qualitative phase could possibly provide further information about the changes learners experienced in their TEI over time. Thus, a mixed-method design can be taken in future endeavors to examine changes in TEI subdomains.

## Data Availability Statement

The raw data supporting the conclusions of this article will be made available by the authors, without undue reservation.

## Ethics Statement

The studies involving human participants were reviewed and approved by Yazd University. The patients/participants provided their written informed consent to participate in this study.

## Author Contributions

TT was involved in the data collection procedure. All authors have analyzed the collected data and contributed the development of the paper.

## Conflict of Interest

The authors declare that the research was conducted in the absence of any commercial or financial relationships that could be construed as a potential conflict of interest.

## Publisher's Note

All claims expressed in this article are solely those of the authors and do not necessarily represent those of their affiliated organizations, or those of the publisher, the editors and the reviewers. Any product that may be evaluated in this article, or claim that may be made by its manufacturer, is not guaranteed or endorsed by the publisher.
